# Supplementary material for: Polyketide synthase gene clusters in the ericoid mycorrhizal fungus Oidiodendron maius: genome-wide prediction, expression profiling, and functional characterization of knockout mutants
Source: Front Plant Sci. 2026 Sep 16;17:1924430. doi: 10.3389/fpls.2026.1924430 (PMC13623903; doi:10.3389/fpls.2026.1924430)
Supplement: Supplementary file 1 [file SupplementaryFile1.zip › Supplementary Materials/Supplementary Folder 1.zip!/Oidma1_AssemblyScaffolds_Repeatmasked/knownclusterblast/cluster45/jgi.p_Oidma1_60487_mibig_hits.html]

| MIBiG Protein | Description | MIBiG Cluster | MiBiG Product | % ID | % Coverage | BLAST Score | E-value |
| --- | --- | --- | --- | --- | --- | --- | --- |
| CAB38889.1 | putative\_salicylate\_hydroxylase\_(putative\_secreted\_protein) | BGC0000315 | Calcium-dependent\_antibiotic\_biosynthetic\_gene\_cluster | 32 | 87.9545454545 | 131 | 3.2e-30 |
| ALV82350.1 | salicylate\_hydroxylase | BGC0001370 | Lipopeptide\_8D1-1\_/\_lipopeptide\_8D1-2\_biosynthetic\_gene\_cluster | 31 | 83.8636363636 | 122 | 1.1e-27 |
| AAT65716.1 | putative\_monooxygenase | BGC0000629 | Aflatrem\_biosynthetic\_gene\_cluster | 27 | 92.0454545455 | 111 | 2e-24 |
| EAU32816.1 | conserved\_hypothetical\_protein | BGC0000160 | Terreic\_acid\_biosynthetic\_gene\_cluster | 26 | 76.8181818182 | 103 | 7e-22 |
| AAT65710.1 | putative\_monooxygenase | BGC0000629 | Aflatrem\_biosynthetic\_gene\_cluster | 27 | 98.1818181818 | 92 | 9.5e-19 |
| AGN71623.1 | salicylate\_hydroxylase | BGC0000027 | Azaphilone\_biosynthetic\_gene\_cluster | 25 | 101.590909091 | 78 | 2.4e-14 |
